# Preaxial Polydactyly of the Foot: Variable Expression of Trisomy 13 in a Case from Central Africa

**DOI:** 10.1155/2014/365031

**Published:** 2014-09-01

**Authors:** Sébastien Mbuyi-Musanzayi, Aimé Lumaka, Bienvenu Yogolelo Asani, Toni Lubala Kasole, Prosper Lukusa Tshilobo, Prosper Kalenga Muenze, François Tshilombo Katombe, Koenraad Devriendt

**Affiliations:** ^1^Department of Surgery, University Hospital, University of Lubumbashi, P.O. Box 1825, Lubumbashi, Democratic Republic of Congo; ^2^Center for Human Genetics, Faculty of Medicine, University of Lubumbashi, P.O. Box 1825, Lubumbashi, Democratic Republic of Congo; ^3^Center for Human Genetics, University Hospitals, KU Leuven, UZ Leuven, Campus Gasthuisberg, Herestraat 49, P.O. Box 602, 3000 Leuven, Belgium; ^4^Department of Pediatrics, University Hospitals, University of Kinshasa, P.O. Box 123, Kin XI, Kinshasa, Democratic Republic of Congo; ^5^Department of Ophthalmology, University Hospital, University of Lubumbashi, P.O. Box 1825, Lubumbashi, Democratic Republic of Congo; ^6^Department of Pediatrics, University Hospital, University of Lubumbashi, P.O. Box 1825, Lubumbashi, Democratic Republic of Congo; ^7^Department of Gynecology, University Hospital, University of Lubumbashi, P.O. Box 1825, Lubumbashi, Democratic Republic of Congo

## Abstract

Trisomy 13 is a chromosomal disorder characterized by a severe clinical picture of multiple congenital anomalies. We here describe the clinical and genetic features and prognosis observed in a newborn with trisomy 13 from Central Africa. He presented the rare feature of preaxial polydactyly of the feet.

## 1. Introduction

Trisomy 13 (also known as Patau syndrome) is the third most common autosomal trisomy [1, 2]. The prevalence is between 1 : 10,000 and 1 : 20,000 live births [3], but it is estimated that the frequency of trisomy 13 is 100 times higher in spontaneous abortions [4, 5]. This chromosomal disorder has a characteristic phenotype consisting of multiple congenital anomalies [6], with a classical clinical triad of microphthalmia or anophthalmia, cleft lip and/or palate, and postaxial polydactyly. However, other anomalies are frequently associated [2, 7]. The objective of this report is to describe the clinical features and prognosis in a Congolese newborn with trisomy 13 and to illustrate the occurrence of a rare manifestation in this syndrome, preaxial polydactyly of the foot.

## 2. Case Report

**Figure 1:**
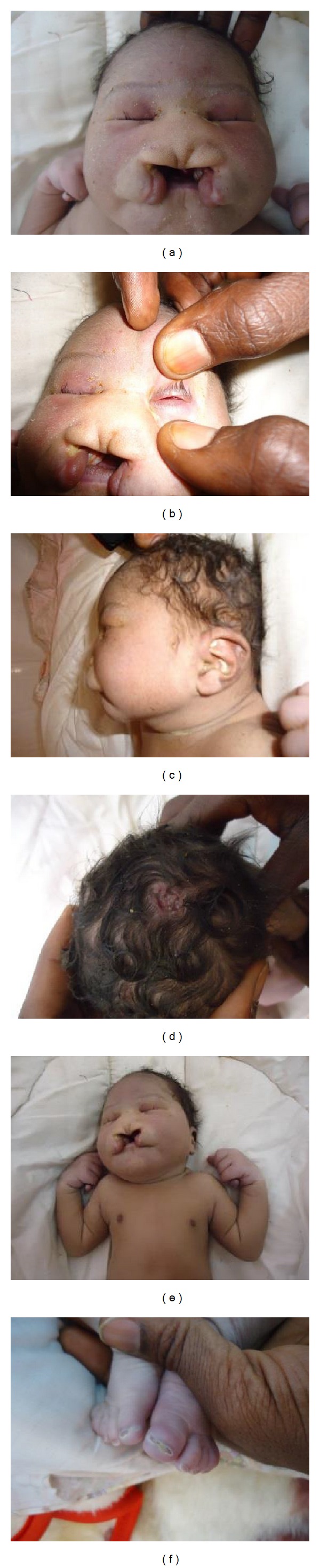
Craniofacial abnormalities observed in the patient. Note (a) median cleft lip and palate, (b) anophthalmia, (c) low-set ears, (d) aplasia cutis/scalp defect, (e) postaxial polydactyly on the hands, and (f) preaxial polydactyly of the feet.

The patient, a male, was referred at an age of two days. He was born at 40 weeks of gestation via a normal spontaneous vaginal delivery with birth weight 3250 g (−0.5 SD). His mother was 25 years old and father was 32 years old; both were healthy and unrelated. Family history was unremarkable. Prenatal ultrasound was not performed. He presented median cleft lip and palate, microcephaly (29 cm–−2.6 SD), bilateral anophthalmia, a posterior scalp defect, short neck, micropenis, and bilateral cryptorchidism (Figure 1). He had bilateral postaxial polydactyly of his hands. Of interest, he also had bilateral preaxial polydactyly of the first toes. The child was hypotonic and died at age of 5 days from acute respiratory distress.

## 3. Methods

Genomic DNA was isolated using standard protocols from the peripheral blood leukocytes and screened for copy number alterations using the Oxford Gene Technology 8 × 60 k Array Platform Custom Design (Catalogue number 027216). Array CGH results were interpreted using Oxford Gene Technology CytoSure Interpret Software_v.3.3.2 (OGT CytoSure, OGT Oxford, UK). All genome coordinates were according to NCBI human genome build 19 (hg19 Feb 2009). We performed array-CGH, which revealed trisomy 13: arr 13q12.11-q34 (20,407,270–115,092,581) ×3 or a duplication of the entire 94.69 Mb of chromosome 13. No additional CNVs were observed. Since karyotyping is not available in this part of the world, we were not able to exclude a Robertsonian translocation.

## 4. Discussion

We here present the clinical and genetic data in newborn with trisomy 13, diagnosed in the Democratic Republic of Congo. Since its first description by Patau in 1960 [8], trisomy 13 has been recognized as one of the three commonly observed autosomal trisomies observed in live newborns, worldwide. In Central Africa, genetics reports on chromosomal imbalances are scarce, and, to the best of our knowledge, there is only one earlier report on trisomy 13 in the Democratic Republic of Congo, dating from the year 1968 [9]. The case we report here presents the classical triad of cleft lip and palate, postaxial polydactyly, and anophthalmia. Each of these features is observed in 60–80% of cases [6, 10]. The patient had a median cleft lip and palate, with marked hypotelorism, characteristic of holoprosencephaly, a common finding in trisomy 13. Brain ultrasound scan could not be performed, since the parents could not afford to pay for it. In addition, the patients presented several additional features, commonly observed in trisomy 13, as shown in Table 1. Postaxial polydactyly (especially of the hands) is reported in 52–70% of cases [6, 11–13]. However, the patient presented a very unusual sign: bilateral preaxial polydactyly of the feet. This finding has been reported twice before [6, 14]. While this may be a coincidence, it is tempting to speculate that the expression of this unusual feature in this Congolese boy may be related to its different genetic background. However, we have no firm evidence to support this at present. Postaxial polydactyly, especially of the hands, is a common feature in Africa, with a reported incidence between 10.4/1000 births in South and Central Africa [15, 16] and 22.78/1000 in Nigeria [17]. In contrast to this, preaxial polydactyly is rare in Central Africa as elsewhere (Table 1). The early death of the patient presented here is not unexpected: the median survival of patients with trisomy 13 varies from 2.5 to 10 days [3, 4, 13, 18]. The probability of survival until one month of age is about 28% and only 5–10% survive for one year [1, 3]. The cause of death may be primary apnea, regardless of the presence of a CNS abnormality [1, 5, 19]. Also, recurrent apnea may be related to the common occurrence of a cyanotic heart defect, pulmonary hypertension, congestive heart failure, aspiration pneumonia, gastroesophageal reflux, laryngomalacia, and seizures [20–22]. The case reported here is the first one with trisomy 13 to be reported in Central Africa (with the exception of a report in 1968) [9]. This probably reflects the current lack of teaching and thus interest and knowledge in human genetics and syndromology [23]. Whereas, in most industrialized countries, trisomy 13 is diagnosed prenatally, the vast majorities of pregnant women in Central Africa currently do not have access to prenatal ultrasound follow-up and are thus confronted with serious emotional distress when facing an unexpected polymalformed newborn. Early clinical recognition of trisomy 13 at birth remains essential to optimize guidance for care of the child and his family. For instance, one can avoid needless and expensive therapeutic or diagnostic interventions, which is crucial in an environment where access to medical care and investigations is difficult and expensive. Moreover, also in this society, an exact diagnosis offers the opportunity to discuss the cause, refute commonly held mystical and traditional beliefs, and relieve misassigned feelings of guilt [24].

## Figures and Tables

**Table 1:** Summary of clinical features in trisomy 13.

Study	Taylor [10]	Hodes et al. [11]	Moerman et al. [12]	Lin et al. [13]	Petry et al. [6]	Quelin et al. [14]	Patient
Year	1968	1978	1988	2007	2013	2014	2014
Country	England	USA	Belgium	Taiwan	Brazil	France	DRC
Samples (*N*)	27	19	12	28	30	3	1
Features	%	%	%	%	%	%	%
Craniofacial							
Abnormal auricles	74	79	25	0	77	0	−
Microphthalmia	70	84	42	54	60	33	−
Anophthalmia	0	11	0	14	10	33	+
Low-set ears	85	0	33	?	47	33	+
Aplasia cutis/scalp defect	0	47	25	29	43	0	+
Microcephaly	59	58	50	61	40	0	+
Cleft palate	67	68	42	?	33	0	+
Cleft lip	56	53	8	?	23	0	+
Short neck	70	16	0	46	30	0	+
Ocular hypotelorism	0	21	17	0	10	0	+
Thorax/abdomen							
Inguinal hernia/umbilical hernia	37	32	−	14	20	0	−
Anogenital							
Cryptorchidism	93	100	50	73	78	0	+
Micropenis	−	5	50	?	30	0	+
Limbs, skin, and neurological							
Postaxial polydactyly hands/feet	70	58	67	64	63	0	+
Single palmar crease	59	0	42	32	33		−
Duplicated hallux	−	−	−	−	3	33	+
Capillary hemangioma	56	37	0	14	27	0	−
Mongolian spot	0	0	0	0	0	0	−
Hypertonia/hypotonia	77	16	0	0	33	0	+
